# Analysis of mitochondrial Complex I activity and ATP hydrolysis capacity of ATP synthase in developing rat brains using frozen tissues

**DOI:** 10.17912/micropub.biology.001641

**Published:** 2025-07-16

**Authors:** Pamela J. Yao, Rachel Munk, Myriam Gorospe, Dimitrios Kapogiannis

**Affiliations:** 1 Laboratory of Clinical Investigation, National Institute on Aging Intramural Research Program, National Institutes of Health; 2 Laboratory of Genetics and Genomics, National Institute on Aging Intramural Research Program, National Institutes of Health

## Abstract

In a previous study, we analyzed the activity of the mitochondrial respiratory Complex II, Complex IV and ATP synthase in frozen tissues of postnatal rat brains (Yao et al., 2023). In this study, we expand our capability of assessing mitochondrial functions using frozen tissue samples. We optimize protocols for measuring the activity of Complex I, and ATP hydrolysis capacity – known as the reverse action – of ATP synthase. We show that the specific functions of these mitochondrial proteins increase linearly as the brain develops.

**Figure 1. Developmental age-dependent increase of mitochondrial respiratory Complex I activity and the ATP hydrolysis capacity of ATP synthase in postnatal rat brains (cortex).**
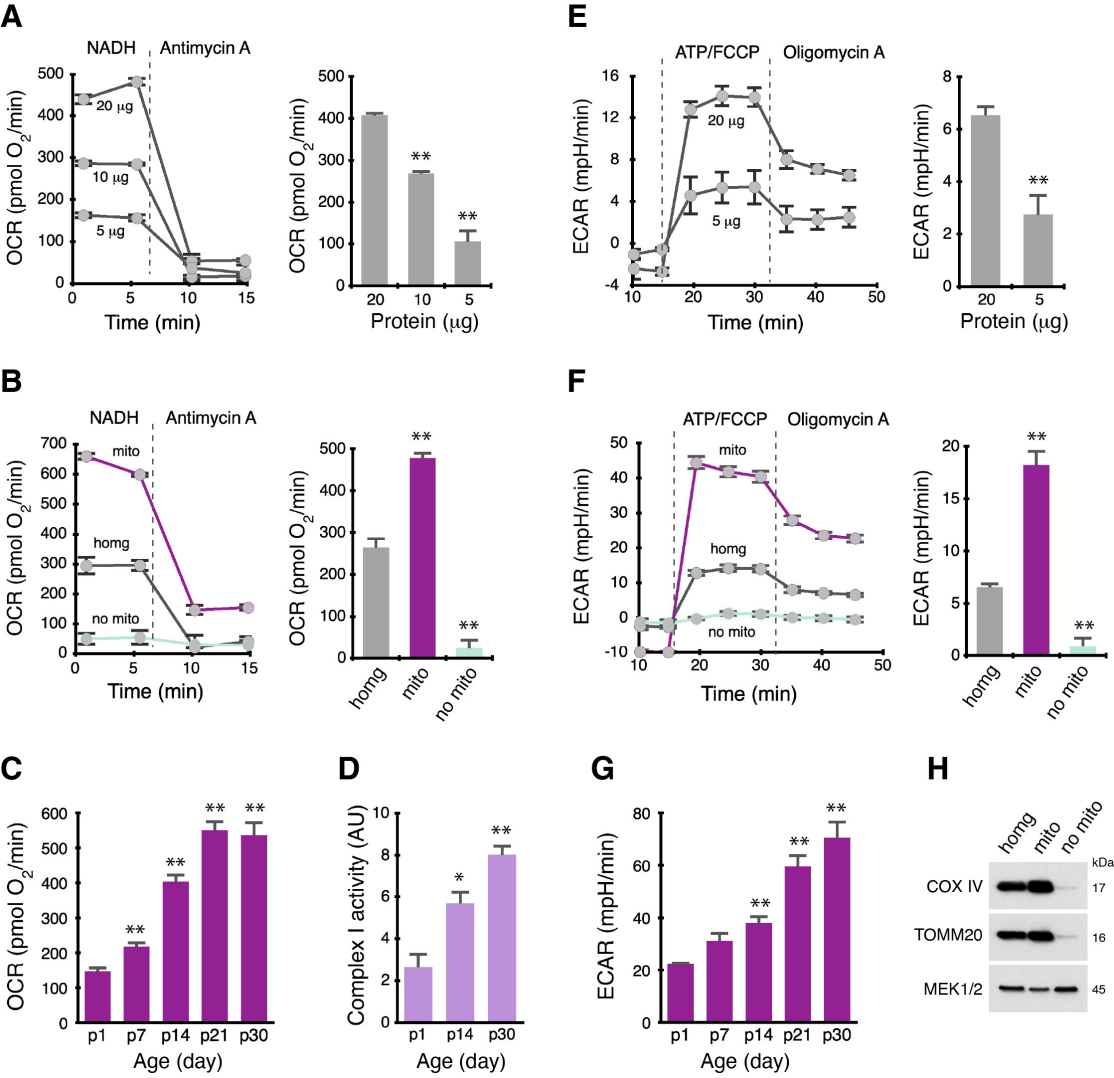
( **A** ) Complex I-mediated respiration in tissue homogenate of frozen rat brains. *Left* , oxygen consumption rate (OCR) traces of NADH-driven respiration. NADH (nicotinamide adenine dinucleotide hydrogen) is the substrate for Complex I. The mean OCR values prior to Antimycin A injection represent the level of Complex I activity. *Right* , Complex I activity level as a function of protein amount in the samples. Data are means ± SEM; n = 3 rats (p30, for postnatal day 30). ( **B** ) Complex I-mediated respiration in tissue homogenate (homg), isolated mitochondria (mito) and mitochondria-free cytosol (no mito) of frozen rat brains. *Left* , OCR traces of NADH-driven Complex I activity. *Right* , the mean OCR level in various cellular fractions as indicated. All samples contained 20 μg of protein. Data are means ± SEM; n = 3 rats (p30). ( **C** ) Levels of Complex I-mediated respiration in mitochondria in the brains of postnatal developing rats. All samples contained 20 μg of protein. Data are means ± SEM; n = 4 rats for all ages. ( **D** ) Colorimetric assay of Complex I activity levels during brain development in postnatal rats. All samples contained 50 μg of protein. Data are means ± SEM; n = 3 rats for all ages. AU, arbitrary unit. ( **E** ) ATP hydrolysis capacity by ATP synthase in tissue homogenate of frozen rat brains. *Left* , traces of extracellular acidification rate (ECAR), a readout of ATP hydrolysis capacity. Adding ATP exogenously forces ATP synthase to work in reverse to hydrolyze ATP. The amount of H ^+^ generated is recorded as ECAR. The mean ECAR values of ATP/FCCP prior to the addition of Oligomycin A represent the ATP hydrolysis capacity of ATP synthase. Oligomycin A is a specific inhibitor of ATP synthase. *Right* , ATP hydrolysis capacity by ATP synthase as a function of protein amount in the samples. ( **F** ) ATP hydrolysis capacity in tissue homogenate (homg), isolated mitochondria (mito) and mitochondria-free cytosol (no mito) of frozen rat brains. *Left* , ECAR traces of ATP hydrolysis activity. *Right* , the mean ECAR level in various cellular fractions as indicated. All samples contained 20 μg of proteins. Data are means ± SEM; n = 3 rats (p30). ( **G** ) ATP hydrolysis capacity of ATP synthase in mitochondria brain development in postnatal rats. All samples contained 20 μg of proteins. Data are means ± SEM; n = 4 rats for all ages. In ( **A** ) and ( **E** ), the values were compared between 20 μg and other amounts. In ( **B** ) and ( **F** ), the values were compared between homogenate and other fractions. In ( **C** ), ( **D** ) and ( **G** ), the values were compared between p1 and other age groups. *p<0.05; **p<0.01, *t* -test. ( **H** ) Immunoblots of various cellular fractions. COX IV and TOMM20, mitochondrial proteins; MEK1/2, a cytosolic protein.

## Description

The proteins of the mitochondrial respiratory chain, from Complex I to IV and ATP synthase, are composed of multiple subunits. Among these respiratory proteins, Complex I comprises the greatest number of subunits, with a total of 45 subunits (in humans) – which, at least in part, could explain why many mitochondrial diseases arise from malfunctioning Complex I subunits (Rodenburg, 2016; Flones et al., 2018; Kverneng et al., 2025). A new study reported that Complex I activity during development controls lifespan (Stefanatos et al., 2025), emphasizing the need for further understanding of Complex I and improving the methods for monitoring and evaluating Complex I.

Immunoblot analysis can reveal the levels of specific Complex I subunits, as exemplified in our previous study (Yao et al., 2023). In the present study we refine and optimize a Seahorse XF-based method to measure the activity of Complex I in frozen tissues. The capability of reliably measuring the activity of Complex I in frozen samples provides a practical means for many future Complex I-focused investigations.

ATP synthase has two functional modes, ATP synthesis and ATP hydrolysis. The commonly accepted dogma is that the membrane potential and the proton gradient dictate the direction of ATP synthase rotation hence its specific actions: the clockwise rotation (forward) of ATP synthase leads to ATP synthesis, whereas counterclockwise rotation (reverse) of ATP synthase, leads to ATP hydrolysis (von Ballmoos et al., 2009; Junge and Nelson, 2015; Weber and Senior, 2000). Given that ATP is the primary energy supply for cells, the simplest assumption is that ATP synthase must be kept in the forward ATP synthesis mode to produce ATP constantly. However, how the two functional modes of ATP synthase are balanced and fine-tuned in different tissues and cell types, as well as in different populations of mitochondria is not fully understood

The activity of ATP synthase can be measured with a commercially available assay (Yao et al., 2023, 2024), although it is unclear if the assay distinguishes the two functional modes of ATP synthase. In the ATP hydrolysis measurement described in the present study, several steps are specifically designed for measuring the reverse mode of ATP synthase. First, addition of Antimycin A blocks respiration, priming ATP synthase to function in its reverse mode. Second, adding ATP in the subsequent step provides ATP synthase with the substrate, thus facilitating the enzyme to hydrolyze ATP. Including FCCP with ATP reduces membrane potential, further ensuring that ATP synthase will operate in its reverse mode. Finally, addition of Oligomycin A blocks ATP synthase’s rotations (both directions), confirming the specificity of the assay.

Quality control pathways of mitochondria are essential in health and lifespan (Fang et al., 2019; Scheibye-Knudsen et al. 2015). Functional deficiencies of mitochondrial have been implicated in a wide range of brain diseases, thus, the development and application of reliable methods to assess mitochondrial functions in frozen post-mortem brain tissue is useful and critically important.

## Methods

*Complex I-mediated respiration measurement*

Oxygen consumption rate (OCR) in the brain tissues (cortex) was measured following a method optimally designed for frozen samples (Acin-Perez et al., 2020; Yao et al., 2023, 2024). The tissue samples were homogenized in ice-cold MAS assay buffer using a pestle motor mixer (Argos). MAS buffer: 70 mM sucrose, 220 mM mannitol, 5 mM KH _2_ PO _4_ , 5 mM MgCl _2_ , 1 mM EGTA, 2 mM HEPES, pH 7.4. The tissue homogenates were centrifuged at 1,000 x *g* for 5 min at 4 ^o^ C, and the supernatants were collected. For isolating mitochondria, the supernatants were centrifuged at 10,000 x *g* for 10 min at 4 ^o^ C. The mitochondrial pellet was re-suspended in MAS buffer, and the supernatants were collected as mitochondria-free cytosol. Protein concentrations of the total homogenates, isolated mitochondria and mitochondria-free cytosol were determined using Pierce BCA protein assay.

The samples containing 5-20 μg of total protein in 70 μl of MAS buffer were added to each well of a Seahorse XF24 cell culture plate (Agilent). After centrifuging the plate at 2,000 x *g* for 5 min at 4 ^o^ C, 455 μl of MAS buffer containing cytochrome c (10 μg/ml), alamethicin (10 μg/ml), and nicotinamide adenine dinucleotide hydrogen (NADH; 1 mM) were added to the wells. NADH, the substrate for Complex I, specifically fuels Complex I-mediated respiration in frozen samples (Acin-Perez et al., 2020; Osto et al., 2020).

Port A of the sensor cartridge plate was loaded with 75 μl of 4 μM Antimycin A. NADH-elicited OCR was measured for 8 min; Antimycin A was then injected and OCR was again measured. Complex I-mediated OCR was calculated by subtracting the Antimycin A-OCR values from the values of NADH-OCR prior to Antimycin A injection.

*ATP synthase-mediated ATP hydrolysis measurement*

The second part of the Complex I-mediated OCR assay described above was continued for assessing the ATP hydrolytic capacity by ATP synthase (Fernandez-del-Rio et al., 2023). Port B and C were loaded with 75 μl of 20 mM ATP and 1 μM FCCP. Port D was loaded with 75 μl of 10 μM Oligomycin A. At the end of the above Antimycin A-OCR measurement, ATP and FCCP were injected from port B and C and the extracellular acidification rate (ECAR) was measured after port C injection. Oligomycin A was then injected to block ATP synthase activity. The maximal ATP synthase’s ATP hydrolytic capacity was calculated by subtracting the Oligomycin A-ECAR values from the ECAR values prior to Oligomycin A injection.

*Complex I activity measurement*

We also used a plate-based colorimetric assay (abcam, #ab109721) to measure Complex I activity in the brain tissues. The brain tissues (cortex) were homogenized in cold PBS and centrifuged at 1000 x *g* for 5 min at 4 ^o^ C. The resultant supernatants were incubated with the assay buffer containing 10% detergent (both were provided in the assay kit) on ice for 30 minutes. After centrifuging at 10,000 x *g* for 10 min at 4 ^o^ C, the supernatants were collected and protein concentrations were determined. The samples containing 50 μg of total protein in 200 μl of the assay buffer were added to the wells of the assay plate. The plate was incubated overnight at 4 ^o^ C followed by the measurement of Complex I activity per the manufacturer’s instruction.

*Immunoblot analysis*

We used standard protocols for immunoblotting. Proteins (20 μg) of various fractions were resolved on 4-12% Bis-Tris NuPAGE gels and transferred to nitrocellulose membranes (0.2 μm pore size). The membranes were then incubated with primary antibodies at 4 ^o^ C followed by species-appropriate, horseradish peroxidase (HRP)-linked secondary antibodies. The bound antibodies were visualized by the enhanced chemiluminescence method (Pierce). Primary antibodies were used that recognized COX IV (#11967, Cell Signaling Technology), TOMM20 (#186734, abcam), and MEK1/2 (#8727, Cell Signaling Technology).

*Statistical analysis*

Statistical analysis was performed using KaleidaGraph (Synergy Software). Groups were compared using unpaired Student’s *t* test. The values represent the means ± SEM from 3-4 independent rat samples.

## Reagents

Sprague-Dawley rats were from Charles River Laboratories; Crl:CD (SD), RRID:RGD_737891, and used as the source of brain cortical tissues. The tissue samples were collected, snap-frozen and stored at -80 ^o^ C. Aliquots of these samples have been used in several previous studies related to developing rat brains (Rivell et al., 2019a,b; Yao et al., 2023). All procedures were approved by the Animal Care and Use Committee of the Intramural Research of the National Institute on Aging, in accordance with the National Research Council for the Care and Use of Laboratory Animals.
