# Erratum: Analysis of mitochondrial Complex I activity and ATP hydrolysis capacity of ATP synthase in developing rat brains using frozen tissues

**DOI:** 10.17912/micropub.biology.001787

**Published:** 2025-08-05

## Description

Erratum: Yao PJ, Munk R, Gorospe M, Kapogiannis D. 2025. Analysis of mitochondrial Complex I activity and ATP hydrolysis capacity of ATP synthase in developing rat brains using frozen tissues. microPublication Biology. 10.17912/micropub.biology.001641.

In the version of the microPublication originally published online, the RRID for Sprague-Dawley rats from Charles River Laboratories was RRID:RGD_737891. The correct RRID is RRID:RGD_734476. This mistake has been corrected online as well as in future PDF versions of this article.

